# Association between Knowledge about Comprehensive Food Education and Increase in Dental Caries in Japanese University Students: A Prospective Cohort Study

**DOI:** 10.3390/nu8030114

**Published:** 2016-02-25

**Authors:** Muneyoshi Kunitomo, Daisuke Ekuni, Shinsuke Mizutani, Takaaki Tomofuji, Koichiro Irie, Tetsuji Azuma, Mayu Yamane, Kota Kataoka, Ayano Taniguchi-Tabata, Hirofumi Mizuno, Hisataka Miyai, Yoshiaki Iwasaki, Manabu Morita

**Affiliations:** 1 Departments of Preventive Dentistry, Okayama University Graduate School of Medicine, Dentistry and Pharmaceutical Sciences, 2-5-1 Shikata-cho, Kita-ku, Okayama 700-8558, Japan; de19013@s.okayama-u.ac.jp (M.K.); pbme8eie@s.okayama-u.ac.jp (S.M.); tomofu@md.okayama-u.ac.jp (T.T.); coichiro@md.okayama-u.ac.jp (K.I.); tetsuji@md.okayama-u.ac.jp (T.A.); de18053@s.okayama-u.ac.jp (M.Y.); de18017@s.okayama-u.ac.jp (K.K.); de19026@s.okayama-u.ac.jp (A.T.-T.); pc2o46pj@okayama-u.ac.jp (Hir.M.); plzs3rog@okayama-u.ac.jp (His.M.); mmorita@md.okayama-u.ac.jp (M.M.); 2 Advanced Research Center for Oral and Craniofacial Sciences, Okayama University Dental School, 2-5-1 Shikata-cho, Kita-ku, Okayama 700-8558, Japan; 3 Health Service Center, Okayama University, 2-1-1 Tsushima-naka, Kita-ku, Okayama 700-8530, Japan; yiwasaki@cc.okayama-u.ac.jp

**Keywords:** food education, dental caries, university students, cohort studies, behavioral science

## Abstract

In Japan, comprehensive food education (*shokuiku*) programs are carried out with the aim of improving dietary practices and thereby reducing the incidence of lifestyle-related diseases, including dental caries. The purpose of this prospective cohort study was to investigate the association between knowledge about *shokuiku* and the increase in dental caries among Japanese university students who had attended a *shokuiku* program while in junior/senior high school. A total of 562 students volunteered to undergo oral examinations over a three-year follow-up period, during which the number of cases of dental caries were recorded. Additional information was collected using a questionnaire survey regarding knowledge about *shokuiku*, dietary habits, and oral health behaviors. In logistic regression analysis, males who lacked knowledge about *shokuiku* had significantly higher odds for dental caries than those who did not (odds ratio (OR), 2.00; 95% confidence interval (CI), 1.12–3.58; *p* = 0.019). On the other hand, among females, those who frequently consumed sugar-sweetened soft drinks had significantly higher odds for dental caries than those who did not (OR, 1.89; 95% CI, 1.05–3.42; *p* = 0.035). These results suggest that having no knowledge about *shokuiku* is associated with a risk of increase in dental caries in Japanese male university students.

## 1. Introduction

The “Food Education Basic Law” (The Basic Law on *Shokuiku*) was enacted by the Japanese government in 2005 with the goal of promoting healthy dietary habits among the Japanese population [1]. After the law was implemented, school-based *shokuiku* programs started in various settings with both high-risk and population approaches. The school-based *shokuiku* programs are performed from preschool to high school, according to the guideline of the Ministry of Education, Culture, Sports, Science and Technology, whereas there are no school-based *shokuiku* programs during university. Students learn about diet and nutrition in their classes. The *shokuiku* program is performed based on the cultural, social and industrial situations in each region. Therefore, there are some differences in the detail methods between the schools. By providing information on appropriate diets, these *shokuiku* programs sought to promote food culture and to improve dietary practices and the food environment and, in turn, to help ensure adequate energy and nutrient intake and prevent lifestyle-related diseases [2].

Good nutrition is essential to promoting overall health and reducing the risk of developing lifestyle-related diseases. Nutritional factors are implicated in many oral and systemic diseases, including dental caries [3]. Dental caries is a lifestyle-related disease known to be associated with inappropriate dietary habits; it remains a common oral problem in many communities. In previous studies, the prevalence of dental caries has been associated with excessive sugar consumption and snacking habits, as well as low socioeconomic status, restricted access to dental services, and ineffective fluoride use [3,4,5,6,7,8], suggesting that unhealthy dietary habits are a risk factor for increase in dental caries.

University students are typically undergoing rapid lifestyle changes due to the transition from living in a home to a school environment, in which they suddenly find themselves responsible for their own health and health-related behavior for the first time. However, it is assumed that students do not seem to generally focus on health promotion efforts due to their youth. As a result, they may develop unhealthy dietary habits. Having knowledge about *shokuiku* may be important in improving unhealthy dietary habits and preventing dental caries among university students.

We previously reported finding an association between *shokuiku* and dental caries in a cross-sectional study [9]. Among university students who had participated in a *shokuiku* program during junior/senior high school, students who lacked knowledge about *shokuiku* had higher odds for experiencing dental caries (decayed, missing and filled teeth (DMFT) > 0) than those who did not [9]. Since this is a cross-sectional study, it remained unclear whether there is a causal relationship in which having knowledge about *shokuiku* could help to prevent dental caries in university students. Therefore, we hypothesized that having knowledge about *shokuiku* at baseline might be able to prevent the increase in dental caries. The aim of this prospective cohort study was to investigate the association between knowledge about *shokuiku* and the increase in dental caries in Japanese university students.

## 2. Experimental Section

### 2.1. Study Population

Of 2184 first-year students who underwent oral examinations (pre-university) and completed questionnaires at the Health Service Center of Okayama University in April 2011, a total of 631 volunteered to undergo oral examinations during a three-year follow-up period until April 2014 (follow-up rate, 28.9%). All students had participated in a *shokuiku* program while in junior/senior high school. After excluding students who returned incomplete questionnaires (*n* = 69), data from 562 students aged 18 to 20 years at baseline were analyzed. This study was approved by the Ethics Committee of Okayama University Graduate School of Medicine, Dentistry and Pharmaceutical Sciences (No. 1039). Verbal consent was obtained from all participants.

### 2.2. Questionnaire

Questionnaires were completed before the health examination at baseline. Participants were asked about their sex and age. Knowledge about *shokuiku* was reported at baseline according to one of the following three responses: (1) I know and can explain the meaning and content of the word “*shokuiku*”; (2) I am only familiar with the word “*shokuiku*”; and (3) I do not know anything about “*shokuiku*” [9]. To clearly distinguish between participants with and without knowledge about *shokuiku*, both responses 2 and 3 were considered to represent having no knowledge about *shokuiku* [9]. Frequency of brushing teeth was measured by using the following the question: “How many times do you brush your teeth daily?” [9]. In addition, the following factors were measured in the form of binary (yes/no) responses: “Have you received regular dental checkups during the past year?”; “Do you use dental floss?”; “Do you use fluoride dentifrices?”; “Do you keep regular mealtimes?”; “Do you frequently snack and/or eat at night?”; and “Do you frequently consume sugar-sweetened soft drinks?” [9,10,11,12].

### 2.3. Oral Examination

Six dentists (Kota Kataoka, Mayu Yamane, Shinsuke Mizutani, Daisuke Ekuni, Koichiro Irie, and Tetsuji Azuma) examined the oral health status of the study participants. The DMFT score (dental caries experience) in the mouth was recorded according to WHO criteria [13]. After the theoretical training, to assess intra- and inter-examiner agreement, DMFT scores were recorded and repeated within a two-week interval in eight volunteers. Data were analyzed using a non-parametric kappa test. The kappa coefficients for intra- and inter-examiner reliability were >0.8.

### 2.4. Statistical Analyses

The participants were divided into two groups based on whether their DMFT score had increased (ΔDMFT > 0) during the three-year follow-up period. Paired t, unpaired t, and chi-square tests were used to determine the presence of significant differences between baseline and re-examination, sex, or the two groups based on knowledge about *shokuiku* or the change in DMFT. In a series of logistic regression models, associations with dental caries were examined, and odds ratios (ORs) and 95% confidence intervals (CIs) were calculated. An increase or no increase in DMFT score during the three-year follow-up period was used as the dependent variable, while knowledge about *shokuiku*, an irregular diet, frequent snacking and/or eating at night, frequent consumption of sugar-sweetened soft drinks, regular dental checkups, brushing teeth (daily frequency), dental floss (usage), fluoride dentifrice (usage) and DMFT score at baseline were used as the independent variables in multivariate analysis. The logistic regression models were reviewed for goodness-of-fit and validated using the Hosmer-Lemeshow statistic.

We estimated sample size based on the nine variables for multivariable logistic regression analysis. For reliable analysis, we required at least 90 events. Because we hypothesized that an incidence of dental caries is 58% by referring to our previous study [9], we needed to include 155 participants.

Because sex is a risk factor for dental caries [14], all analyses were stratified by sex. A test for a statistical interaction between sex and knowledge about *shokuiku* was conducted in the multivariate analysis.

A *p*-value < 0.05 was considered to be significant. The statistical program SPSS (version 23.0; IBM, Tokyo, Japan) was used for data analyses.

## 3. Results

Significant differences were observed in mean DMFT scores (±standard deviation (SD)) between baseline and re-examination in both males (1.9 ± 2.8 *vs.* 2.7 ± 3.3) and females (2.2 ± 2.8 *vs.* 3.3 ± 3.4), respectively (*p* < 0.001). Overall, 191 participants (34.0%) reported that they knew and could explain the meaning and content of *shokuiku*.

Table 1 shows the results of sex differences in knowledge about *shokuiku*, dietary habits, and oral health behaviors. Significant differences were found in some parameters (knowledge about *shokuiku*, an irregular diet, frequent consumption of sugar-sweetened soft drinks, brushing teeth) between males and females (*p* < 0.05).

Table 2 shows the association between knowledge about *shokuiku*, dietary habits, and oral health behaviors. Those who lacked knowledge about *shokuiku* snacked and/or ate at night more frequently, regardless of sex.

A significantly higher percentage of males in the group with no increase in DMFT had knowledge about *shokuiku* compared with the group that had an increase in DMFT (*p* = 0.020) (Table 3). Among females, a significantly higher percentage in the group with an increase in DMFT reported frequently consuming sugar-sweetened soft drinks compared with the group with no increase in DMFT (*p* = 0.035) (Table 3).

In logistic regression analyses, males who had no knowledge about *shokuiku* had significantly higher odds for an increase in dental caries than those who did (*p* = 0.019) (Table 4). In females, no significant association was found between knowledge about *shokuiku* and an increase in DMFT score. On the other hand, the odds for an increase in dental caries in females were found to be significantly related to the frequent consumption of sugar-sweetened soft drinks (*p* = 0.035).

## 4. Discussion

*Shokuiku* programs aim to improve dietary practices so as to help prevent lifestyle-related diseases [2]. In our previous cross-sectional study, we found an association between knowledge about *shokuiku* and the experience of dental caries as a lifestyle-related disease [9]. However, to the best of our knowledge, this is the first prospective cohort study investigating the association between knowledge about *shokuiku* and the increase in dental caries in Japanese university students. After adjusting for potential confounders, male Japanese students at Okayama University who had no knowledge about *shokuiku* were found to have significantly higher odds of an increase in dental caries than those who did. This finding suggests that poor knowledge about *shokuiku* may lead to an increase in dental caries.

In addition to biological processes [15], non-biological or socio-behavioral risk factors for dental caries have been reported [16,17]. Recently, empirical attention has shifted to the relationship between dental caries and broader ecological influences such as education [8]. Although the exact role of *shokuiku* in the etiology of dental caries remains unclear, several underlying mechanisms have been proposed. Participation in *shokuiku* programs led to significantly lower consumption of snack foods high in sugar among primary school children [18], and significantly decreased consumption of soft drinks among college students [12]. Since sugar consumption directly affects the prevalence of dental caries [19,20], *shokuiku*, especially knowledge about snack food and sugar intake, may promote improved dietary habits, less sugar intake, and a lower prevalence of dental caries [9,12]. In the univariate analysis (Table 2), male participants did not show any significant relation between knowledge of *shokuiku* and dietary habits. However, males who had no knowledge about *shokuiku* snacked and/or ate at night more frequently (1.5 times, *p* = 0.054), and actually had higher adjusted ORs for the increase in dental caries than those who had knowledge (Table 4); these results support our hypothesis.

On the other hand, no significant relationship was observed between knowledge about *shokuiku* and dental caries in females. This might be explained by the fact that females had better health behaviors than males regarding sugar intake and tooth brushing (Table 1), which could lessen the effect of knowledge about *shokuiku* on the increase in dental caries. However, further investigation is required to clarify the relationship between knowledge about *shokuiku* and the increase in dental caries in females.

In females, a significant relationship was found between the odds for increase in dental caries and frequent consumption of sugar-sweetened soft drinks. In a previous study, after controlling for confounders, children with the highest intake of sugar-sweetened soft drinks were 2.0 to 4.6 times more likely to have severe dental caries than those with the lowest intake [21]; this finding also supports our hypothesis.

*Shokuiku* programs in Japan and food education in other countries have led to improved dietary practices such as snacking habits [1]. Evaluating and enhancing knowledge about *shokuiku* at regular health examinations might be a useful approach to prevent dental caries, especially for male university students, who tend to develop poor dietary habits during the period of transition from the home to the school living environment [22].

In this study, no relationship was found between the increase in dental caries and the use of fluoride dentifrice. This finding was inconsistent with that from a previous review [23]. The reason for this may have been the very low usage rate of fluoride dentifrice (20.2% in males and 23.0% in females) in this study, which was remarkably lower than the recent market share of fluoridated dentifrices in Japan (about 90%) [24]. It is possible that many students do not actually know whether they use fluoride dentifrice [9,25].

In this study, mean DMFT scores (±SD) at baseline and re-examination were 2.1 ± 2.8 and 3.1 ± 3.4, respectively, in the university students aged 18 to 24 years. Although the sample size, decade, and race in the present study differed from those in previous studies comprising young adults aged 15 to 24 years [26,27], the mean DMFT scores were within the same range. However, the mean DMFT scores in this study were lower than those reported in a Japanese national survey of dental diseases in 2005 (3.2 ± 3.9 for those aged 15–19 years; 5.9 ± 4.8 for those aged 20–24 years) [28]. This difference may be explained by differences in education level [29].

Our study did have some limitations. First, since the follow-up rate was low, a selection bias may have been present; this could lead to an over- or under-estimation of the true relationship. In this study, a statistically significant difference in DMFT scores at baseline was found between participants who were and were not followed up (2.6 ± 3.2 *vs.* 2.1 ± 2.8, respectively) (*p* < 0.001). The participants who were followed up may have been healthier than the whole population; therefore, the results of this single study should be interpreted with some caution. Second, we did not consider possible confounders such as bacterial factors [15], salivary factors [15], social capital [30,31] or socioeconomic status [8,17,32,33] in this study. Future studies are needed to assess the effects of these factors. Third, all participants were recruited from among students at Okayama University, which may limit the ability to extrapolate these findings to the general population of young people. Fourth, we did not confirm that knowing about *shokuiku* correctly reflected actual dietary habits. It is possible that one student may state knowing about the *shokuiku* program without having information, whereas another student may state not being familiar with the terminology but may still have knowledge about healthy dietary habits. Finally, we could not investigate the change in confounding variables during the three years, which may be a potential source of bias. However, there are no school-based *shokuiku* programs during university, which could lessen the effect of later exposure on outcome.

## 5. Conclusions

The findings of this prospective cohort study suggest that having no knowledge about *shokuiku* is associated with the risk of increase in dental caries in Japanese male university students.

## Figures and Tables

**Table 1:** Sex differences in knowledge about comprehensive food education (*shokuiku*), dietary habits, and oral health behaviors at baseline.

Covariate	Male	Female	Total	*p* Value ^†^
	(*n* = 262)	(*n* = 300)	(*n* = 562)	
Knowledge about *shokuiku*				
	75 (28.6) *	116 (38.7)	191 (34.0)	0.012
An irregular diet				
	73 (27.9)	56 (18.7)	129 (23.0)	0.01
Frequent snacking and/or eating at night				
	55 (21.0)	79 (26.3)	134 (23.8)	0.138
Frequent consumption of sugar-sweetened soft drinks				
	74 (28.2)	62 (20.7)	136 (24.2)	0.036
Regular dental checkups				
	31 (11.8)	48 (16.0)	79 (14.1)	0.156
Brushing teeth (daily frequency)				
≥2 times	207 (79.0)	274 (91.3)	481 (85.6)	<0.001
Dental floss (usage)				
	12 (4.6)	14 (4.7)	26 (4.6)	0.961
Fluoride dentifrice (usage)				
	53 (20.2)	69 (23.0)	122 (21.7)	0.427

Notes: * *n* (%); ^†^ Chi-square test, compared to males.

**Table 2:** Association between knowledge about comprehensive food education (*shokuiku*), dietary habits, and oral health behaviors.

Covariate	Male		*p* Value ^†^	Female		*p* Value ^†^
	Knowledge	Knowledge		Knowledge	Knowledge	
	(+)	(−)		(+)	(−)	
	(*n* = 75)	(*n* = 187)		(*n* = 116)	(*n* = 184)	
An irregular diet						
	19 (25.3) *	54 (28.9)	0.563	23 (19.8)	33 (17.9)	0.682
Frequent snacking and/or eating at night						
	10 (13.3)	45 (24.1)	0.054	21 (18.1)	58 (31.5)	0.010
Frequent consumption of sugar-sweetened soft drinks						
	17 (22.7)	57 (30.5)	0.204	21 (18.1)	41 (22.3)	0.384
Regular dental checkups						
	6 (8.0)	25 (13.4)	0.224	16 (13.8)	32 (17.4)	0.408
Brushing teeth (daily frequency)						
≥2 times	57 (76.0)	150 (80.2)	0.449	108 (93.1)	166 (90.2)	0.387
Dental floss (usage)						
	7 (9.3)	5 (2.7)	0.020	5 (4.3)	9 (4.9)	0.816
Fluoride dentifrice (usage)						
	15 (20.0)	38 (20.3)	0.953	31 (26.7)	38 (20.7)	0.224

Notes: * *n* (%); ^†^ Chi-square test.

**Table 3:** Differences in knowledge about comprehensive food education (*shokuiku*), dietary habits, and oral health behaviors between the DMFT groups.

Covariate	Male		*p* Value ^†^	Female		*p* Value ^†^
	DMFT	DMFT		DMFT	DMFT	
	No Increase	Increase		No Increase	Increase	
	(*n* = 138)	(*n* = 124)		(*n* = 147)	(*n* = 153)	
Knowledge about *shokuiku*						
	48 (34.8) *	27 (21.8)	0.020	50 (34.0)	66 (43.1)	0.105
An irregular diet						
	38 (27.5)	35 (28.2)	0.901	26 (17.7)	30 (19.6)	0.67
Frequent snacking and/or eating at night						
	25 (18.1)	30 (24.2)	0.228	38 (25.9)	41 (26.8)	0.852
Frequent consumption of sugar-sweetened soft drinks						
	32 (23.2)	42 (33.9)	0.055	23 (15.6)	39 (25.5)	0.035
Regular dental checkups						
	17 (12.3)	14 (11.3)	0.797	24 (16.3)	24 (15.7)	0.880
Brushing teeth (daily frequency)						
≥2 times	115 (83.3)	92 (74.4)	0.070	137 (93.2)	137 (89.5)	0.261
Dental floss (usage)						
	7 (5.1)	5 (4.0)	0.668	6 (4.1)	8 (5.2)	0.638
Fluoride dentifrice (usage)						
	30 (21.7)	23 (18.5)	0.521	29 (19.7)	40 (26.1)	0.187

Notes: DMFT: decayed, missing and filled teeth; * *n* (%); ^†^ Chi-square test.

**Table 4:** Odds ratios regarding knowledge about comprehensive food education (*shokuiku*), dietary habits, and oral health behaviors.

Independent Variables	Male (*n* = 262)		Female (*n* = 300)	
	Adjusted OR *	*p* Value	Adjusted OR *	*p* Value
	(95% CI)		(95% CI)	
Knowledge about *shokuiku*				
Yes	1		1	
No	2.00 (1.12–3.58)	0.019	0.66 (0.40–1.07)	0.090
An irregular diet				
No	1		1	
Yes	0.88 (0.50–1.56)	0.657	1.08 (0.58–2.01)	0.813
Frequent snacking and/or eating at night				
No	1		1	
Yes	1.09 (0.57–2.08)	0.799	1.05 (0.61–1.82)	0.958
Frequent consumption of sugar-sweetened soft drinks				
No	1		1	
Yes	1.41 (0.79–2.51)	0.243	1.89 (1.05–3.42)	0.035
Regular dental checkups				
Yes	1		1	
No	1.29 (0.59–2.86)	0.525	1.02 (0.53–1.97)	0.945
Brushing teeth (daily frequency)				
≥2 times	1		1	
<2 times	1.72 (0.90–3.27)	0.098	1.55 (0.66–3.63)	0.318
Dental floss (usage)				
Yes	1		1	
No	0.90 (0.26–3.06)	0.863	0.69 (0.21–2.23)	0.690
Fluoride dentifrice (usage)				
Yes	1		1	
No	1.28 (0.68–2.41)	0.453	0.71 (0.40–1.24)	0.221
DMFT	1.04 (0.95–1.14)	0.348	0.94 (0.86–1.02)	0.121

Notes: OR: odds ratio; CI: confidence interval; DMFT: missing and filled teeth; * Adjusted for knowledge about *shokuiku*, an irregular diet, frequent snacking and/or eating at night, frequent consumption of sugar-sweetened soft drinks, regular dental checkups, tooth brushing (daily frequency), dental floss (usage), fluoride dentifrice (usage), and DMFT scores at baseline. *P*-value for interaction was 0.018.
